# Demand for community-based care services and its influencing factors among the elderly in affordable housing communities: a case study in Nanjing City

**DOI:** 10.1186/s12913-020-5067-0

**Published:** 2020-03-23

**Authors:** Tiantian Gu, Jingfeng Yuan, Lingzhi Li, Qiuhu Shao, Chuanjun Zheng

**Affiliations:** 1 School of Civil Engineering, Southeast University, Jiangning District, Nanjing, 211189 China; 2 School of Civil Engineering, Nanjing Tech University, Nanjing, 211816 China

**Keywords:** Affordable housing community, The elderly, Community-based care, Demand, Determinant

## Abstract

**Background:**

Community-based care services refers to the professional services provided at home to the elderly with formally assessed demands. The growth of the elderly population has increased the demand for these services, and this issue is even worse in the affordable housing community (AHC) of China. Understanding of elderly’s demands for different types of community-based care services and its determinations would enable the implementation of appropriate incentive schemes to promote utilization of community-based care services in the AHCs of China.

**Methods:**

Guided by previous studies, a conceptual framework was developed. Then, a questionnaire was designed and a community based survey was conducted from May 10–20, 2018 in Daishan AHC of Nanjing City, China. Four hundred eight participants from 25,650 elderly people were selected by systematic random sampling technique. Binary logistic regression was applied to the data about the elderly’ primary demands for community-based care services in the AHC, to quantify the elderly’s demands and explore related individual-level factors.

**Results:**

The finding indicates that more than 50% of respondents had the demand for an elderly care hotline, building health archives, on-call nursing and doctor visits, medical lectures, regular medical examinations and sporting fitness. The binary logistic regression models revealed that the primary demands of the elderly for community-based care services were influenced by distinct factors.

**Conclusions:**

Our findings help clarify different types of community-based care services and provide fresh information about the demand for community-based care among the elderly in AHCs. Several policy implications are discussed to enhance the efficiency of community-based care service provision.

## Background

By the end of 2018, China’s elderly population aged 60 years and over was 249 million (17.9% of the total population), making it the only country in the world with an elderly population of more than 200 million. More seriously, the number of elderly people will continue to grow at a pace of over 5 million per year in the next 3 years [1, 2]. To address the severe aging problem in China, community-based care, defined as professional care provided at home to the elderly with formally assessed demands, has been advocated and become an increasingly significant mode of care provision [3, 4]. For instance, in ‘the 13^th^ five year national plan (2016-2020) for developing the elderly care system’ issued in 2017, it is required to give priority to the development of community-based care services [5]. Nevertheless, recent studies have illustrated that many studies paid more attention on improving the accessibility of elderly facilities than on the utilization of community-based care related to the demand side [6–8]. For increasing demands of the elderly for different types of services, the lack of supply has been the main barrier to developing the elderly care system [9–12].

This issue is even worse in the affordable housing community (AHC) of China, which is one kind of residential areas. To address the growing housing problems for low-income individuals in China, many AHCs were vigorously advocated between 2011 and 2015 with the characteristics of the remote location and low-income population aggregation [13–15]. In particular, the proportion of the elderly in these communities is higher than in other residential communities [16]. For example, by the end of 2017, the proportion of the elderly in the Daishan AHC (one of the AHCs in Nanjing) had reached 30%, which far exceeded the overall proportion of the elderly in Nanjing (20.85%) [5]. Due to these innate characteristics of the AHCs, the demands for community-based care services in these communities are rapidly increasing while the quantity of these services is seriously inadequate [17].

Previous studies on the AHCs mainly focus on the allocation of elderly healthcare facilities [5], impacts of indoor facilities management on the quality of life of the elderly [18], social impact assessment of affordable housing projects [15], assessment of elderly-adaptability level [19]. There are limited studies concerning the demand of the elderly for community-based care services in the AHCs of China. Meanwhile, some scholars explored the demand for health care services or elderly care services in specific areas of different countries [6, 20–23]. Various factors that influence the demand for community-based care services have been taken into consideration and certain contributions have been made. It is found that the demand for community-based care services, at the individual level, is mainly influenced by four categories of factors: (a) differences in the demographic structure, such as age, gender, educational level, health status; (b) family structure; (c) the availability of social and material resources, such as income source, monthly income, source of medical expenditure; and (d) elderly care intention [24–26]. For example, to examine four types of factors related to the demand of the elderly living in the cities for community-based care services, the logistic model was used and it was found that female, the elderly aged 80 and over, with a higher educational level, living alone, with higher income, and the elderly who preferred to receive elderly care in long-term care institutions had higher demands for medical care services [22, 27]. Additionally, the determinants of demands for the elderly care vary across different cultural and environmental settings [28, 29].

However, many challenges in community-based care provision have been identified in previous studies. First, the provision of community-based care services for elderly people are generally diverse across different countries due to different historical paths of development, population structure, welfare systems and culture [17]. Few studies systematically summarized the types of community-based care services, especially those in China. Second, there is a lack of quantitative research on the elderly’s actual demands for various types of community-based care services. Third, few scholars have explored the determinants of the elderly’ demands for these services and the distinctions between these determinants.

To promote the development of community-based care worldwide and particularly in the AHCs of China, it is of great significance to conduct research on the demand of the elderly for various community-based care services in the AHCs. Moreover, the efficiency of the care provision could be improved when taking the elderly’s characteristics into account. Hence, this study aims to explore the demands of the elderly and their determinants for different types of community-based care services in the AHCs of China. The specific research questions are as follows:

1. What are the elderly’ actual demands for different types of community-based care services in the AHCs of China?
2. What are the determinants of the primary demands of the elderly for the services in the AHCs?
3. And are there any differences between the influencing factors of the elderly’s demands for different services in the AHCs?

## Methods

There are currently no standard validated surveys analyzing the demands of the elderly for community-based care services in the AHCs of China. To quantify such demands and investigate associated individual variables, a conceptual framework and quantitative survey were developed. Then, the analysis was carried out in three steps. First, descriptive statistical analysis was conducted with the data. Second, the demands of the elderly for community-based care services in Daishan AHC were analyzed. Third, significant variables which were related to the primary demands were explored respectively. A detailed description of the framework and survey is provided below.

### Conceptual framework

In general, community-based care services can be divided into four categories as follows: assistance with activities of daily life service (AADLS), medical care service (MCS), cultural and entertainment service (CES), psychological and legal service (PLS) [17, 30, 31]. Table 1 provides a summary of various types of community-based care services for the elderly and describes the specific contents of these services.

**Table 1:** A summary of various types of community-based care services for the elderly

Category	Type	Brief description	Researchers
Assistance with activities of daily living service (AADLS)	1. The elderly care hotline	Providing support, information, advice, or a referral for the elderly through the telephone hotline.	[20, 32–34]
	2. Meal-aid	Providing canteens or centralized meal delivery service.	
	3. Clean-aid	Providing indoor cleaning services and specialized cleaning services for the elderly.	
	4. Bath-aid	Providing the visiting bath service for the elderly to help them take a bath.	
	5. Walk-aid	Providing scheduled transportation services.	
	6. Daycare	Providing day care and support services to frail elders aged 60 and above who are lacking care of family members during day time.	
Medical care service (MCS)	1. Building health archives	Helping the elderly in the community to establish and maintain health archives.	[34–36]
	2. On-call nursing and doctor visits	Helping the elderly save time and money with 24/7 access to a doctor by phone or online video anytime, anywhere. Doctors offer a diagnosis, treatment options and prescription, if medically necessary.	
	3. Rehabilitation therapy	Providing care that can help the elderly get back, keep, or improve abilities that they need for daily life. These abilities may be physical, mental, and/or cognitive.	
	4. Medical lecture	Providing education on lifestyle, nutrition and disease management.	
	5. First-aid	Providing emergency response to unexpected serious health events and unexpected security incidents, such as offering assistance to the elderly suffering sudden cardiovascular and cerebrovascular diseases.	
	6. Regular medical examinations	It is a common form of preventive medicine involving visits to a general practitioner by well feeling elderly on a regular basis.	
	7. Medication Guide	Medication reminders and supervision.	
Cultural and entertainment service (CES)	1. Chess and mahjong	Providing the elderly with places to play chess and mahjong in the community.	[27, 33, 37–39]
	2. Drama, singing and dancing	Providing the elderly with places to watch the drama, sing and dance in the community.	
	3. Calligraphy and painting	Providing the elderly with places to do calligraphy and painting in the community.	
	4. Daily reading	Providing the elderly with places to read books daily in the community, such as the reading room.	
	5. Sporting fitness	Providing the elderly with places to exercise in the community.	
	6. Learning activities	Providing the elderly with places to conduct learning activities in the community.	
	7. The elderly tourism	Providing tourism services for the elderly.	
Psychological and legal service (PLS)	1. Chat-aid	Providing emotional support, ideological communication, and relieve their emotional loneliness by chatting with the elderly face to face.	[22, 34, 40, 41]
	2. Psychological counseling	Providing mental health and adjustment services to the elderly and also consultation with them (Providers are professional counselors).	
	3. Legal aid	Providing legal services in civil matters to the elderly.	
	4. Daily mental care	Providing relieve mental disorders, and alleviate mental stress for the elderly to meet their daily mental needs (Providers are volunteers).	
	5. Mediation	Helping the elderly to mediate with others by expert consultation.	

**Fig. 1:**
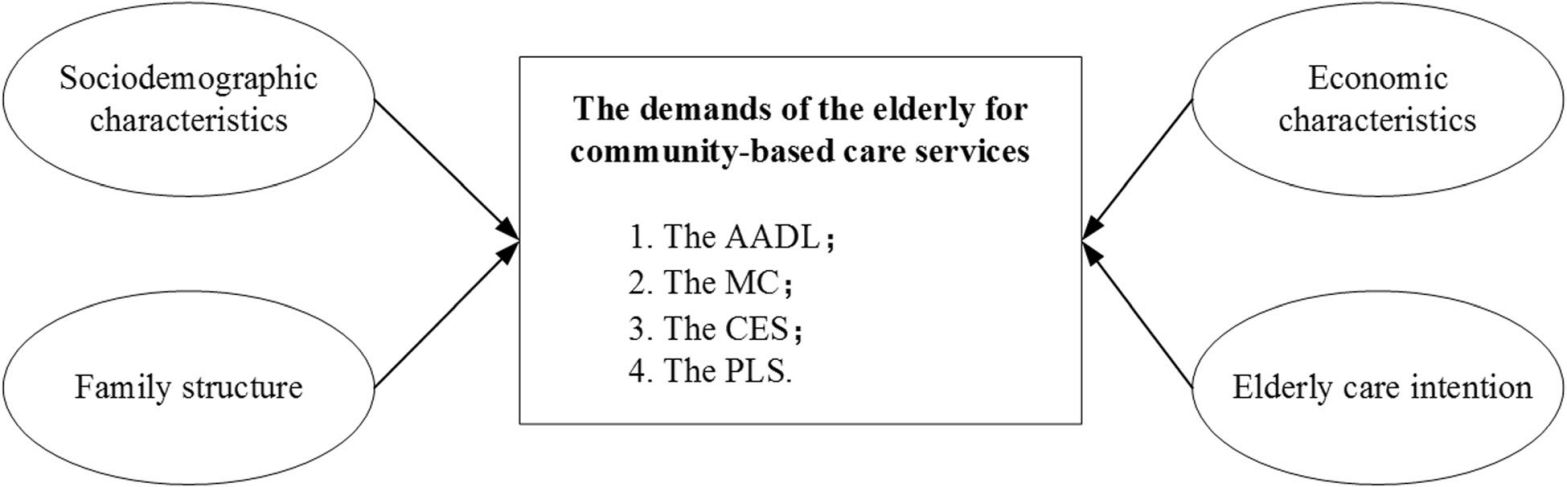
Conceptual framework of this study

According to previous research works, sociodemographic characteristics, family structure, economic characteristics, and elderly care intention are generally factors at individual levels that influence the elderly’s demands for community-based care services [24, 25, 27]. On this basis, an extensive conceptual framework has been developed for testing the factors which influence the elderly’s demands for various types of community-based care services in the AHCs (Fig. 1).

### Questionnaire design

Four categories of community-based care services are identified, guided by current literature. In this study, 25 types of community-based care of four categories are selected as dependent variables and evaluated by responses to the question: ‘Do you have a demand for this type of community-based care services?’. The response variable of the respondents *Y*_*i*_ was measured as a dichotomous variable with possible values: *Y*_*i*_ = 1, if the respondent demanded for this type of community-based care and *Y*_*i*_ = 0 otherwise.

Based on the above conceptual framework, combined with the innate characteristics of the AHCs, potential factors expected to be correlated with the demand for community-based care services are included as independent variables in the study. They are sociodemographic characteristics (gender, age, educational level, health status, whether suffering from chronic diseases, self-care ability, career before retirement), family structure (living status), economic characteristics (monthly income, income source, source of medical expenses) and elderly care intention (location of preference to receive elderly services, e.g. private homes, community-based care facilities and long-term care institutions). The questionnaire in Additional file 1 was used to collect the data used in the study.

### Data collection

As a city in eastern China, Nanjing is located in Jiangsu Province. Daishan AHC in Nanjing City was selected as the study area for two reasons. The principal reason is that the proportion of the elderly in Daishan AHC is much higher than the overall population of the elderly in Nanjing. Another reason is that urgent demand has been caused for community-based care services due to the lack of elderly facilities [5]. In 2017, the number of elderly people living in Daishan AHC was nearly 30,000. Hence, the statistical theoretical sample size in Daishan AHC was approximately 379 with a 95% confidence level and a 5% significance level. Due to multiple deviations in the survey, 420 questionnaires were distributed to the elderly people over 60 years old in this community from May 10–20, 2018. Random sampling was used to ensure that the number of observations from each community was proportional. To achieve high response rates and reliable results, face-to-face interviews were used to collect the data. Fourteen students from ‘Livelihood Security Research Center’ at our university participated in conducting the survey. After the face-to-face interviews, 408 valid questionnaires were returned and the rate of effective recovery of the questionnaires was 97.14%.

### Statistical model

Binary logistic regression was applied for the model estimation since the dependent variable is a dummy: coded 0 (non-demand for this type of community-based care services) or 1 (demand for this type of community-based care). The specific statistical model is as follows:

1 $$ \ln \left(\frac{P_j}{1-{P}_j}\right)=\alpha +\sum \limits_{i=1}^k{\beta}_i{x}_i+\gamma \ast f+\sum \limits_{i=1}^n{\lambda}_i{z}_i+\mu \ast w+\varepsilon $$

Where, *j* (j = 1, 2, …,25) is one of community-based care services; *P*_*j*_ is the probability of the elderly’s demand for *j*^*th*^ of community-based care services in the AHCs; $$ \frac{P_j}{1-{P}_j} $$ is the ‘odds ratio’; $$ \ln \left(\frac{P_j}{1-{P}_j}\right) $$$$ \ln \left(\frac{P_j}{1-{P}_j}\right) $$ is the log odds ratio, or ‘logit’; *α* is a random constant term; *x*_*i*_ (i = 1,2, …,7) represents the sociodemographic characteristics of the elderly; *f* represents the family structure of the elderly; *z*_*i*_ (i = 1,2,3) represents the economic characteristics of the elderly; *w* represents the wishes for the community-based elderly care; *β*_*i*_, *γ*, *λ*_*i*_ and *μ* are the corresponding independent variable coefficients; *ε* is the random error term. SPSS 22.0 statistical software was used for data processing and regression analysis.

In order to explore the relationship between the likelihood that having demand for a specific service and the factors a bit more, the services that over half of the respondents needed were selected as dependent variables and several binary logistic models were fitted to the data using the predictor variables (sociodemographic characteristics, family structure, economic characteristics, elderly care intention).

## Results

As shown in Additional file 1, the survey collected information about respondents’ sociodemographic characteristics, family structure, economic characteristics, elderly care intention, and their demands for 25 types of community-based care services. Analysis of the information was performed below.

### Descriptive statistics of the respondents

Table 2 presents individual characteristics of the respondents. In terms of sociodemographic characteristics, the respondents were predominately female, older people (older than 70 years), low education level, general health status, suffering from chronic diseases, having self-care ability, daily laborer before retirement (agriculture, forestry, animal husbandry or fishing worker). Nearly half of the respondents lived with their spouses and 25.50% of the respondents lived with their children. In terms of economic characteristics, the respondents whose income were less than 1000 yuan accounted for 35.80%, followed by the respondents whose income was more than 2500 yuan (29.90%), which means that the income of the elderly was low and the polarization was severe. In addition, 58.30% of the respondents relied on retired pension and endowment insurance, while only 2.90% of them relied on personal labor income. 55.40% of those surveyed were covered by health insurance, and only 18.60% were dependent upon their children for support, showing that most elderly do not rely on their kids to receive income or pay medical expenses. With respect to elderly care intention, 72.10% of the respondents chose to receive community care services in private homes, and only 1.50% of them were willing to receive these services in long-term care institutions. This indicates that receiving care in private homes and the community was still the most optimal option for the elderly in the AHCs, with the long-term care institutions the least favorable. The outcomes of this survey are consistent with the actual situation.

**Table 2:** Simple descriptive statistics of the sample

Variables		Frequency	Percentage (*N* = 408)
**Sociodemographic characteristics**			
Gender	Male	156	38.20%
	Female	252	61.80%
Age	60–64	50	12.30%
	65–69	62	15.20%
	70–74	98	24.00%
	75–79	88	21.60%
	80 and above	110	27.00%
Educational level	Illiteracy	124	30.40%
	Primary school	108	26.50%
	Middle school	104	25.50%
	Technical secondary school or High school	56	13.70%
	Junior College	10	2.50%
	Undergraduate or above	4	1.00%
Health status	Very good	116	28.40%
	General	204	50.00%
	Bad	88	21.60%
Whether suffering from chronic diseases	No	88	21.60%
	Yes	320	78.40%
Self-care ability	Fully self-care	290	71.10%
	Partial self-care	90	22.10%
	Without self-care ability	28	6.90%
Career before retirement	Daily laborer	182	44.60%
	Civil servant	2	0.50%
	Public institution employee	28	6.90%
	State-owned company employee	88	21.60%
	Private company employee	52	12.70%
	Merchant	34	8.30%
	Other	22	5.40%
**Family structure**			
Living status	Living alone	68	16.70%
	With spouse	198	48.50%
	With children	104	25.50%
	With grandchildren	8	2.00%
	Three generations living together	30	7.40%
**Economic characteristics**			
Monthly income	< 1000 RMB	146	35.80%
	1000–1500 RMB	60	14.70%
	1500–2000 RMB	40	9.80%
	2000–2500 RMB	40	9.80%
	> 2500 RMB	122	29.90%
Income source	Pension and endowment insurance	238	58.30%
	Money from children or relatives	96	23.50%
	Personal labor income	12	2.90%
	Government aid	42	10.30%
	Other	20	4.90%
Source of medical expenses	Self-pay	102	25.00%
	Medical insurance	226	55.40%
	Children aid	76	18.60%
	Other	4	1.00%
**Elderly care intention**			
Elderly care intention	Private homes	108	72.10%
	Long-term care institutions	6	1.50%
	Community-based care facilities	294	26.50%

### The demand for community-based care services of the elderly in Daishan AHC

**Fig. 2:**
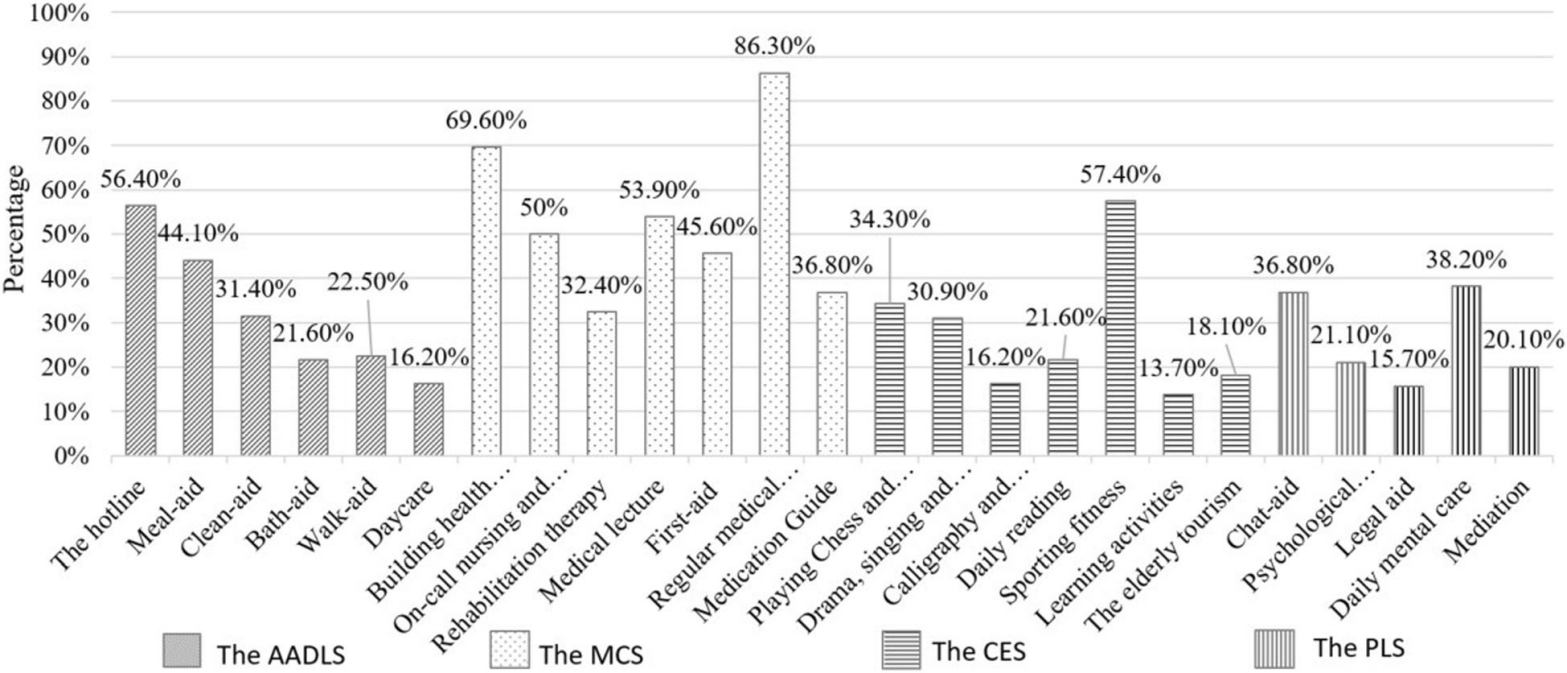
The expressed demand for 25 types of community-based care services by the respondents

According to the survey, it is indicated that respondents had the highest demand for the MCS, while their demands for other services vary from high to low. Moreover, over half of the respondents demanded for the elderly care hotline in the AADLS, building health archives in the MCS, on-call nursing and doctor visits in the MCS, medical lecture in the MCS, regular medical examinations in the MCS, and sporting fitness in the CES. The proportion of the respondents’ demands for each type of community-based care services are shown in Fig. 2.

In terms of demands for the AADLS, the respondents had the highest demand (56.40%) for the elderly care hotline, while the demand for meal-aid (44.10%), clean-aid (31.40%), bath-aid (21.60%), walk-aid (22.50%) and day care (16.20%) were reduced respectively. As for the MCS, a higher demand (86.30%) for regular medical examinations was recorded, followed by building health archives (69.60%), medical lecture (53.90%) and on-call nursing and doctor visits (50.00%). It could be seen that the respondents pay much attention to their daily health status. With regard to the CES, the demand for sporting fitness was higher, accounting for 57.40%, while playing chess and mahjong, drama, singing and dancing, calligraphy and painting, daily reading, learning activities and the elderly tourism respectively accounts for 34.30, 30.90, 16.20, 21.60, 13.70 and 18.10%. The demand for outdoor sports fitness activities was significantly higher than that of indoor activities. In terms of the PLS, the demand for daily mental care and chat-aid was higher, accounting for 38.20 and 36.8% respectively. In other words, the highest demand for the PLS is related to communication, e.g. daily mental care, psychological counseling and chat-aid, which means the elderly in the AHCs have a certain demand for spiritual care.

### Results of binary logistic regression analysis

#### Assessment of model fit

The Hosmer and Lemeshow test was applied to assess goodness of fit of these logistic regression models, which uses a Pearson test statistics to compare the observed and the fitted counts [42, 43]. The null hypothesis H_0_ (the model provides a good fit) and alternative hypothesis H_1_ (the model does not fit the data) were tested respectively. Except for the model for the medical lecture (the fit index of this model was 0.03), the *p* value of the Hosmer and Lemeshow test exceeded 0.05 for the remaining five models, which can be seen in Table 3. Thus, there is no evidence to reject the hypothesis H_0_ for the elderly care hotline (Y1), building health archives (Y2), on-call nursing and doctor visits (Y3), regular medical examinations (Y4), and sporting fitness (Y5). However, the model related to the medical lecture did not show good fit for the data. So its regression results were not included in Table 3.

**Table 3:** Logistic regression results of the main demands for community-based care services

Variables	The elderly care hotline	Building health archives	On-call nursing and doctor visits	Regular medical examinations	Sporting fitness
	OR	OR	OR	OR	OR
**Sociodemographic characteristics**					
Gender (Male)	1.308	0.577	1.230	0.396*	1.224
Educational level	1.116	1.327	1.391*	1.068	1.004
Health status	1.141	1.257	0.545**	0.918	2.545***
Self-care ability	0.710	0.415**	0.643	0.488	0.912
Career (other)	1.000	1.000	1.000	1.000	1.000
Daily laborer	0.381	0.518	0.167**	0.762	1.126
Public institution employee	0.445	0.599	0.098**	0.58	0.327
State-owned company employee	0.387	0.372	0.170*	0.320	0.243*
Private company employee	0.775	0.946	0.075***	0.619	0.487
Family structure					
Living status (Three generations living together)	1.000	1.000	1.000	1.000	1.000
With spouse	2.701*	0.722	0.322*	2.297	3.221*
With children	2.986*	1.029	0.695	4.011*	3.349*
**Economic characteristics**					
Income source (other)	1.000	1.000	1.000	1.000	1.000
Retirement pension and endowment insurance	1.057	6.030**	3.385	8.878*	0.363
Money from children or relatives	0.736	8.729**	4.476*	1.393	0.338
Personal labor income	0.432	2.074	11.666*	4.061	0.144*
**Elderly care intention**					
Elderly care intention (Community-based care facilities)	1.000	1.000	1.000	1.000	1.000
Private homes	0.255***	0.408*	0.648	0.687	1.114
Long-term care institution	1.462	0.032**	0.194	0.386	0.377
Constant	2.427	0.000	75.722*	10.360	0.074
**The*p*value of Hosmer and Lemeshow Test**	0.098	0.186	0.276	0.243	0.210
**Percentage of correct prediction**	68%	77%	71%	84%	71%

1.The category in parentheses for each variable is the reference group; 2. *** *p* < 0.001, ** *p* < 0.01, * *p* < 0.05; 3. Table S1 in Additional file 2 describes confidence interval data of these five models

#### Validation of predicted probabilities

**Fig. 3:**
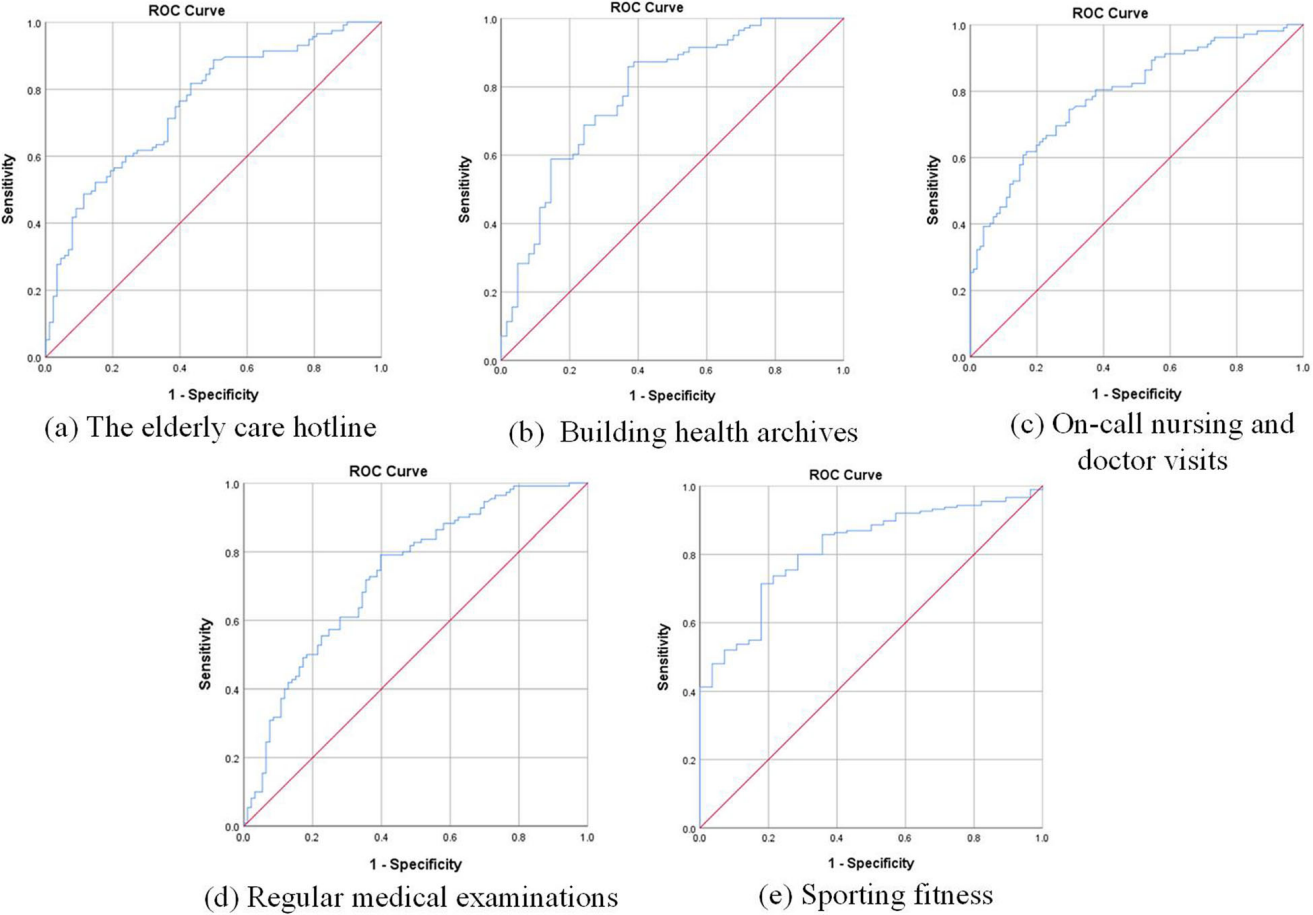
The ROC curves for initial demands and predicted probabilities taken together

The classification table demonstrates the consistency of the predicted probabilities with the actual outcomes [44]. According to the percentage of correct prediction in Table 3, the correct predictions for the Y1-Y5 were 68, 77, 71, 84, 71%, more than 60%, respectively. It shows that the prediction ability of these five models was good. Furthermore, the ROC curve was applied to evaluate the predictive power, which is a sensitivity plot against 1- specificity for all possible thresholds [42]. As an accepted performance metric, the higher area under the ROC represents the model’s better predictive capacity. Figure 3 shows the ROC curves for original demands and predicted probabilities taken together of the five models. All areas under the curves were more than 0.730, verifying good predictive ability of the models from another aspect.

#### Interpretation of logistic regression coefficients

Based on the results of model fit statistics and validation of predicted probabilities, five binary logistic models related to the Y_1_-Y_5_ were finally determined (Table 3). The results displayed in Table 3 reveal that gender, educational level, health status, self-care ability, career before retirement, living status, income source, and elderly care intention were found to be significantly associated with the elderly’s five primary demands for the community-based care services (*p* < 0.05).

As for the elderly’ demands for the elderly care hotline, this kind of demand was significantly affected by living status (*p* < 0.05) and elderly care intention (*p* < 0.001). Specifically, respondents who lived with their spouse (OR = 2.701) and children (OR = 2.986) were more likely to have this demand than those who lived with their children and grandchildren (Three generations living together). However, respondents who lived alone had no significant difference in such demand with those who lived with their children and grandchildren (*p* > 0.05). In addition, respondents who preferred to receive elderly care in private homes were 77.50% (0.225–1, OR = 0.255) less likely to have this demand than those who preferred to receive elderly care in community-based care facilities.

In terms of the elderly’ demands for building heath archives, this demand was significantly associated with self-care ability (*p* < 0.05), income source (*p* < 0.01), and elderly care intention (*p* < 0.01). Respondents with poor self-care ability were found to be 0.415 times (OR = 0.415) less likely to have such demand. Though respondents who depended on personal labor income did not have a substantial distinction in this demand with those who received economic income through other channels, respondents whose financial source were from retirement pension and endowment insurance and money from children or relatives were 6.030 (OR = 6.030) and 8.729 (OR = 8.729) times more likely to have this demand respectively. Besides, respondents who preferred to receive elderly care in private homes and long-term care institutions were 59.20% (0.408–1, OR = 0.408) and 96.80% (0.032–1, OR = 0.032) less likely to have this demand than those who preferred to receive elderly care in community-based care facilities.

As for the elderly’s demands for on-call nursing and doctor visits, this demand was significantly associated with educational level, health status, career before retirement, living status, and income source. Respondents who had higher demand were positively related to educational level (OR = 1.391) and negatively related to health status (OR = 0.545). The respondents, who worked in the field of agriculture, forestry, animal husbandry and fishing (OR = 0.167), as well as those who worked in public institutions (OR = 0.098) or whom worked in state-owned companies (OR = 0.170) and private companies (OR = 0.075) before they retired, were less likely to have a demand for on-call nursing and doctor visits than others. Respondents who lived with their spouse were 67.80% less likely (0.322–1, OR = 0.322) to have such demand than those who lived with their children and grandchildren (Three generations living together). Respondents whose financial source were from children or relatives (OR = 4.476) and personal labor income (OR = 11.666) were 4.476 and 11.666 times more likely to have a demand for on-call nursing and doctor visits respectively.

Taking a look at the elderly’s demands for regular medical examinations, this demand was significantly associated with gender, living status, and income source. The odds of respondents who had this demand were negatively related to the male (OR = 0.396). Respondents who lived with their children were 4.011 times (OR = 4.011) more likely to have such demand than those who lived with their children and grandchildren (Three generations living together). Besides, higher demand for regular medical examinations was positively related to the respondents who received financial support from retirement pension and endowment insurance (OR = 8.878).

In terms of the elderly’s demands for sporting fitness, our regression results show that such demand was significantly associated with health status, career before retirement, living status, and income source. Respondents who had poor health status were found to be 2.545 times (OR = 2.545) more likely to have such demand. The respondents who worked in state-owned companies before they retired were 75.7% less likely (0.243–1, OR = 0.243) to have demand for sporting fitness than others. Besides, the respondents, who lived with their spouse, as well as those who lived with their children, were 3.221 times (OR = 3.221) and 3.349 times (OR = 3.349) more likely to have this demand than those who lived with their children and grandchildren (Three generations living together). Surprisingly, the respondents whose financial source were from personal labor income were 85.6% less likely (0.144–1, OR = 0.144) to have demand for sporting fitness.

## Discussion

By analyzing the survey data, the elderly’ actual demands for different types of community-based care services in the AHCs of China are quantified. Then, based on the conceptual framework, five binary logistic models were fitted to the primary demands and corresponding influencing factors were explored separately. We can be confident of some significant findings.

First, the primary demands of the elderly in the AHCs are regular medical examinations in the MCS, building health archives in the MCS, sporting fitness in the CES, the elderly care hotline in the AADLS, medical lectures in the MCS, and on-call nursing or doctor visits in the MCS. It can be seen that the elderly in the AHCs have a higher demand for medical care services but a lower demand for psychological and legal services. One possible explanation is that the income of the elderly in the AHCs is generally low and thus their safety needs lower down in Maslow’s hierarchy have not yet been satisfied [45]. More seriously, insufficient provision of elderly healthcare facilities, poor accessibility of existing facilities, and lack of professional services have been the main barriers to the supply of medical care services due to the innate characteristics of the AHCs [5]. Similarly, the elderly in low-income countries tend to have less access to health services than those in high-income countries [46]. Therefore, medical care services and assistance with activities of daily living services should be prioritized and guaranteed in such communities.

Second, sociodemographic characteristics influence the primary demands of the elderly for community-based care in the AHCs, and these effects differ between services. The binary logistic regression analysis revealed that gender only affects regular medical examinations, but it has no effect on other services. It could be interpreted that women are more concerned about their health and they usually have higher demand for health monitoring [17]. The elderly with high education level are more likely to have a demand for on-call nursing and doctor visits while education level does not affect other demands. This result is not surprising because the educated elderly may be more aware of the value of modern medicine [6, 47]. Similarly, the improvement of self-care capacity of the elderly only increases the likelihood of having demand for building health archives. It is reported that the elderly with good self-care ability tend to build health records so that they can resort to community health care centers when they feel unwell [22]. Career before retirement is proved to have impacts on the elderly’ demands for on-call nursing or doctor and sporting fitness. For example, state-owned company employees are less likely to demand these two types of services. This is because that the demand for elderly care can be relatively guaranteed for the elderly who have stable occupations [33]. In conjunction with the study above, the supply of community-based care in the AHCs should take the sociodemographic characteristics of the elderly into consideration.

Third, family structure has an effect on the elderly’ demands for the elderly care hotline, on-call nursing and doctor visits, regular medical examinations, and sporting fitness in the AHCs, but does not affect their demand for building health archives. Precisely, the elderly who live with their spouses are more likely to be in demand for the elderly care hotline and the sporting fitness, while their demand for on-call nursing and doctor visits is less likely to happen. In addition, the elderly who live with their children are more likely to have demands for the elderly care hotline, regular medical examinations, sporting fitness. With the nuclear family now becoming China’s main social interaction, family support systems and intergenerational relationships are weakening, especially in the AHCs [48, 49]. Consequently, older people living with their spouses rely more on the elderly care hotline and exercise more to be fit. Notably, older couples are normally able to travel in groups, so demand for on-call nursing and doctor visits is less probable to occur [27]. Meanwhile, modernization, urbanization and the weakening of filial piety are leading to the migration of large numbers of young adults to work [50, 51]. Though the elderly live with their children, they also need information about the community-based care services, frequent physical examinations, and fitness, just as the elderly who live with their spouses, to ensure their quality of life.

Fourth, economic characteristics influence all primary demands of the elderly for community-based care in the AHCs except for their demand for the elderly care hotline. Generally, the elderly care hotline is free in China and thus the income source does not affect the elderly’s demand for such service [22]. In more concrete terms, their demand for regular medical examinations is comparatively high for the elderly whose income source is retirement pension/endowment insurance, labor income and money from children/relatives. The reason is that these elderly have a certain financial support to obtain this service, particularly in comparison to the lower income group [6]. It is noteworthy that the elderly with labor income are less likely to demand sporting fitness because they tend to think positively about their perceived health status [52, 53]. Therefore, the economy characteristic determines the quality of life of the elderly directly.

Fifth, elderly care intention impacts the elderly’s demand for the elderly care hotline, building health archives, and on-call nursing and doctor visits in the AHCs. Specifically, the elderly who preferred to receive care in private homes or long-term care institution are less likely to have demands for these three types of services. The possible explanation is that the elderly who tend to receive elderly care at home or in institutions hold negative attitudes towards community-based care services, which means that the elderly care intention determines the attention and recognition of community-based care [27, 33].

## Conclusions

Understanding the elderly’s demand for community-based care in the AHCs not only helps improve the efficiency of the care provision, but also helps to develop the elderly care industry and thus enhance the living standards of the elderly. This paper aims to generate fresh information about the elderly’ demands for community-based care in the AHCs. A conceptual framework was developed to measure the elderly’s demands for community-based care in the AHCs and investigate their determinants. Binary logistic analysis was implemented on 408 data surveyed from Daishan AHC to quantify the elderly’s demands and explore related individual-level factors. The empirical findings indicate that the elderly care hotline in the AADLS, building health archives in the MCS, on-call nursing and doctor visits in the MCS, medical lecture in the MCS, regular medical examinations in the MCS, and sporting fitness in the CES were the primary services demanded by the elderly. Except for the binary logistic regression model for medical lecture, the other five models for the primary services show good fit for the data. The in-depth analysis of these five models reveals that the primary demands of the elderly for community-based care services were influenced by distinct factors.

According to these empirical results, specific policy implications can be obtained. First, the government should pay attention to the elderly’s demands for community-based care services in the AHCs. The introduction of a market competition mechanism is essential in order to enrich the content of community-based care and improve the community-based care system in which government, communities and families are involved. Second, the sociodemographic characteristics of the elderly should be considered in the supply of community-based care services. To enable the elderly and their relatives to choose the relevant services under their current circumstances, service providers should tailor the supply of community-based supports according to the needs of certain elderly subgroups. Third, given the living status of the elderly in the AHCs, more care should be taken to the elderly. It is necessary to publicize the notion of ‘respect and care for the elderly’ in the AHCs and to implement relevant policies to encourage family members to engage in the provision of community-based care. Fourth, policy on income support could be introduced to enhance the elderly’ purchasing power for community-based care services, especially those with lower income. The steps that can be taken are to improve the subsidy mechanism of community-based care and provide some financial support for low-income elderly in the AHCs. In addition, publicity to the community-based care system should be given to improve the understanding and acceptance of community-based care services among the elderly.

This research contributes to enrich the study of community-based care and the AHC. On the one hand, it clarifies different types of community-based care services and provides fresh information about the demand for community-based care among the elderly in the AHCs by evaluating their actual demand for various services. On the other hand, it enables policymakers understand the determinants of the elderly’ primary demands for the services in the AHCs of China and make proper decisions to enhance the efficiency of community-based care service provision. Moreover, more extended models could be developed based on this conceptual framework and applied to the demand for elderly care services in other countries. However, this research has two limitations. First, the results need to be carefully explained due to the current sample size. Future research should consider examining the elderly’ demands with different types of community-based care services in the AHCs of China when larger-scale sets of data are available. Second, the expressed demand of the elderly and their determinants may vary across different areas or counties. It may be interesting to include other factors in the conceptual framework, such as variables related to the politics, economy and culture, when designing strategies for other areas and counties.

## Supplementary information

**Additional file 1:** Questionnaire on demands of the elderly for community-based care services in affordable housing communities. This is the questionnaire that was used to collect the data used in the study.

**Additional file 2: Table S1**. Logistic regression results of the main demands for community-based care services (with confidence intervals data), describes the regression coefficients and confidence intervals data of these five models.

## Data Availability

The data that support the findings of this study are available from the Nanjing Civil Affairs Bureau of China but restrictions apply to the availability of these data, which were used under license for the current study, and so are not publicly available.

## Abbreviation

AADLS: Assistance with activities of daily living service
AHC: Affordable housing community
CES: Cultural and entertainment service
MCS: Medical care service
PLS: Psychological and legal service
