# Supplementary material for: Demand for community-based care services and its influencing factors among the elderly in affordable housing communities: a case study in Nanjing City
Source: BMC Health Serv Res. 2020 Mar 23;20:241. doi: 10.1186/s12913-020-5067-0 (PMC7092588; doi:10.1186/s12913-020-5067-0)
Supplement: Supplementary file 1 — Additional file 1: Questionnaire on demands of the elderly for community-based care services in affordable housing communities. This is the questionnaire that was used to collect the data used in the study. [file 12913_2020_5067_MOESM1_ESM.pdf]

Questionnaire on demands of the elderly for community-based care services in affordable housing communities

Dear Sir/madam,

Hello, we are researchers from the Livelihood Security Research Center of Southeast University. We want to know about the demands of the elderly for community-based care services in affordable housing communities. I hope you can spare a little time to complete this questionnaire, and thank you for your participation!

Section 1: Basic Information

1. What's your gender?

A. Male

B. Female

2. What is your age?

A. 60-64 years old

B. 65-69 years old

C. 70-74 years old

D. 75-79 years old

E. 80 years old and above

3. What is your education level?

A. Illiterate

B. Primary school

C. Middle school

D. High school (secondary school)

E. Junior college

F. Bachelor degree or above

4. What is your health condition?

A. Good

B. General

C. Bad

5. Do you suffer from chronic diseases? (Such as hypertension, diabetes, arthritis, heart disease, etc.)

A. Yes

B. No

6. How is your self-care ability?

A. Completely able to take care of themselves

B. Partially unable to take care of themselves

C. Completely unable to take care of themselves

7. What was your occupation before your retirement?

A. Agriculture, forestry, animal husbandry or fishing worker (Daily laborer)

B. Civil servant

C. Public institution employee

D. State-owned company employee

E. Private company employee

F. Merchant

G. Other

8. How do you live?

A. Living alone

B. With your spouse

C. With children

D. With grandchildren

F. Three generations living together

9. What is your disposable income for a month?

A. within 1000 RMB

B. 1000-1500 RMB

C. 1500-2000 RMB

D. 2000-2500 RMB

E. above 2500 RMB

10. What is your main source of income?

A. Pension and endowment insurance

B. Money from children or relatives

C. Personal labor income

D. Government aid

E. Other

11. Who pays for your medical expenses?

A. Self-pay

B. Medical insurance

C. Children aid

D. Other

12. Where do you want to get elderly services?

A. Private homes

B. Long-term care institutions

C. Community-based care facilities

Section 2: Demand for community-based care services

13. Which of the following community-based care services do you need? (Tick the box if you need this service)

Category	Type	Demand
Assistance with activities of daily living service (AADLS)	1. The elderly care hotline	
	2. Meal-aid	
	3. Clean-aid	
	4. Bath-aid	
	5. Walk-aid	
	6. Daycare	
Medical care service (MCS)	1. Building health archives	
	2. On-call nursing and doctor visits	
	3. Rehabilitation therapy	
	4. Medical lecture	
	5. First-aid	
	6. Regular medical examinations	
	7. Medication Guide	
Cultural and entertainment service (CES)	1. Chess and mahjong	
	2. Drama, singing and dancing	
	3. Calligraphy and painting	
	4. Daily reading	
	5. Sporting fitness	
	6. Learning activities	
	7. The elderly tourism	
Psychological and legal service (PLS)	1. Chat-aid	
	2. Psychological counseling	
	3. Legal aid	
	4. Daily mental care	
	5. Mediation	
