# Supplementary material for: Demand for community-based care services and its influencing factors among the elderly in affordable housing communities: a case study in Nanjing City
Source: BMC Health Serv Res. 2020 Mar 23;20:241. doi: 10.1186/s12913-020-5067-0 (PMC7092588; doi:10.1186/s12913-020-5067-0)
Supplement: Supplementary file 2 — Additional file 2: Table S1. Logistic regression results of the main demands for community-based care services (with confidence intervals data), describes the regression coefficients and confidence intervals data of these five models. [file 12913_2020_5067_MOESM2_ESM.pdf]

Table S1 Logistic regression results of the main demands for community-based care services (with confidence intervals)

Variables	Y_1	Y_2	Y_3	Y_4	Y_5
	OR (95% CI)	OR (95% CI)	OR (95% CI)	OR (95% CI)	OR (95% CI)
**Sociodemographic characteristics**					
Gender(Male)	1.308(0.760-2.249)	0.577(0.316-1.055)	1.230(0.699-2.167)	0.396(0.175-0.894)*	1.224(0.714-2.098)
Educational level	1.116(0.848-1.470)	1.327(0.976-1.805)	1.391(1.052-1.840)*	1.068(0.712-1.603)	1.004(0.779-1.292)
Health status	1.141(0.765-1.701)	1.257(0.813-1.945)	0.545(0.361-0.824)**	0.918(0.495-1.701)	2.545(1.686-3.841)***
Self-care ability	0.710(0.441-1.143)	0.415(0.227-0.759)**	0.643(0.396-1.044)	0.488(0.193-1.230)	0.912(0.589-1.413)
Career (other)	1.000	1.000	1.000	1.000	1.000
Daily laborer	0.381(0.117-1.240)	0.518(0.126-2.121)	0.167(0.044-0.640)**	0.762(0.163-3.563)	1.126(0.359-3.534)
Public institution employee	0.445(0.091-2.164)	0.599(0.102-3.522)	0.098(0.020-0.479)**	0.580(0.060-5.560)	0.327(0.077-1.389)
State-owned company employee	0.387(0.113-1.333)	0.372(0.093-1.494)	0.170(0.044-0.663)*	0.320(0.057-1.792)	0.243(0.073-0.814)*
Private company employee	0.775(0.209-2.869)	0.946(0.196-4.571)	0.075(0.018-0.316)***	0.619(0.100-3.850)	0.487(0.141-1.684)
**Family structure**					
Living status (Three generations living together)	1.000	1.000	1.000	1.000	1.000

With spouse	2.701(1.028-7.098)*	0.722(0.263-1.980)	0.322(0.122-0.848)*	2.297(0.739-7.135)	3.221(1.318-7.872)*
With children	2.986(1.105-8.066)*	1.029(0.368-2.875)	0.695(0.259-1.860)	4.011(1.171-13.735)*	3.349(1.306-8.586)*
**Economic characteristics**					
Economic source(other)	1.000	1.000	1.000	1.000	1.000
Retirement pension and endowment insurance	1.057(0.308-3.621)	6.030(1.593-22.825)**	3.385(0.860-13.319)	8.878(1.477-53.371)*	0.363(0.090-1.468)
Money from children or relatives	0.736(0.216-2.505)	8.729(2.263-33.665)**	4.476(1.133-17.678)*	1.393(0.312-6.222)	0.338(0.085-1.341)
Personal labor income	0.432(0.078-2.387)	2.074(0.346-12.425)	11.666(1.440-94.497)*	4.061(0.422-39.078)	0.144(0.024-0.861)*
**Elderly care intention**					
Elderly care intention (Community-based care facilities)	1.000	1.000	1.000	1.000	1.000
Private homes	0.255(0.134-0.483)***	0.408(0.195-0.853)*	0.648(0.360-1.169)	0.687(0.257-1.833)	1.114(0.625-1.987)
Long-term care institution	1.462(0.190-11.269)	0.032(0.003-0.339)**	0.194(0.020-1.878)	0.386(0.037-4.030)	0.377(0.053-2.667)
Constant	2.427	0.000	75.722*	10.360	0.074
**The p value of Hosmer and Lemeshow Test**	0.098	0.186	0.276	0.243	0.210
**Percentage of correct prediction**	68%	77%	71%	84%	71%

Notes: 1. Y_1: The elderly care hotline, Y_2: Building health archives, Y_3: On-call nursing and doctor visits, Y_4: Regular medical examinations, Y_5: Sporting fitness; 2.The category in parentheses for each variable is the reference group; 3. *** p<0.001, ** p<0.01, * p<0.05.
